# RIZ2 at the crossroad of the EGF/EGFR signaling in colorectal cancer

**DOI:** 10.1186/s12967-023-04621-6

**Published:** 2023-10-18

**Authors:** Marzia Di Donato, Erika Di Zazzo, Annamaria Salvati, Carmela Sorrentino, Giorgio Giurato, Donatella Fiore, Maria Chiara Proto, Monica Rienzo, Amelia Casamassimi, Patrizia Gazzerro, Maurizio Bifulco, Gabriella Castoria, Alessandro Weisz, Giovanni Nassa, Ciro Abbondanza

**Affiliations:** 1 Department of Precision Medicine, University of Campania “Luigi Vanvitelli”, Naples, Italy; 2 Department of Medicine and Health Sciences “V. Tiberio”, University of Molise, Campobasso, Italy; 3 Laboratory of Molecular Medicine and Genomics, Department of Medicine, Surgery and Dentistry ‘Scuola Medica Salernitana’, University of Salerno, 84081 Baronissi, Italy; 4 CRGS-Genome Research Center for Health, University of Salerno Campus of Medicine, 84081 Baronissi, Italy; 5 Department of Pharmacy, University of Salerno, Fisciano, Italy; 6 Department of Environmental, Biological, and Pharmaceutical Sciences and Technologies, University of Campania “Luigi Vanvitelli”, Caserta, Italy; 7 Department of Molecular Medicine and Medical Biotechnologies, University of Naples “Federico II”, Naples, Italy

**Keywords:** *PRDM2*, *RIZ2* overexpression, Colorectal cancer, EGFR pathway, ZD1839, RNA-seq

## Abstract

**Background:**

Colorectal cancer (CRC) is the third most deadly and fourth most diagnosed cancer worldwide. Despite the progress in early diagnosis and advanced therapeutic options, CRC shows a poor prognosis with a 5 year survival rate of ~ 45%. *PRDM2/RIZ*, a member of PR/SET domain family (*PRDM*), expresses two main molecular variants, the PR-plus isoform (*RIZ1*) and the PR-minus (*RIZ2*). The imbalance in their expression levels in favor of *RIZ2* is observed in many cancer types. The full length RIZ1 has been extensively investigated in several cancers where it acts as a tumor suppressor, whereas few studies have explored the RIZ2 oncogenic properties. *PRDM2* is often target of frameshift mutations and aberrant DNA methylation in CRC. However, little is known about its role in CRC.

**Methods:**

We combined in-silico investigation of The Cancer Genome Atlas (TCGA) CRC datasets, cellular and molecular assays, transcriptome sequencing and functional annotation analysis to assess the role of RIZ2 in human CRC.

**Results:**

Our in-silico analysis on TCGA datasets confirmed that *PRDM2* gene is frequently mutated and transcriptionally deregulated in CRC and revealed that a *RIZ2* increase is highly correlated with a significant *RIZ1* downregulation. Then, we assayed several CRC cell lines by qRT-PCR analysis for the main *PRDM2* transcripts and selected DLD1 cell line, which showed the lowest *RIZ2* levels. Therefore, we overexpressed *RIZ2* in these cells to mimic TCGA datasets analysis results and consequently to assess the *PRDM2/RIZ2* role in CRC. Data from RNA-seq disclosed that *RIZ2* overexpression induced profound changes in CRC cell transcriptome via EGF pathway deregulation, suggesting that RIZ2 is involved in the EGF autocrine regulation of DLD1 cell behavior. Noteworthy, the forced *RIZ2* expression increased cell viability, growth, colony formation, migration and organoid formation. These effects could be mediated by the release of high EGF levels by *RIZ2* overexpressing DLD1 cells.

**Conclusions:**

Our findings add novel insights on the putative RIZ2 tumor-promoting functions in CRC, although additional efforts are warranted to define the underlying molecular mechanism.

**Supplementary Information:**

The online version contains supplementary material available at 10.1186/s12967-023-04621-6.

## Background

The human PRDMs (PRDF1 and RIZ1 homology domain containing proteins), members of a kruppel-like zinc finger subfamily, share a conserved PR/SET domain, followed by zinc finger domains [1–3]. Through regulation of the chromatin architecture, several PRDMs modulate gene expression, by either recognizing specific consensus sequences in promoters or acting as non-DNA binding cofactors [1–3]. Particularly, some PRDMs are endowed with histone methyltransferase activity [1–3]. PRDMs are involved in many developmental processes and play key roles in cell differentiation as well as in transducing several signaling pathways [1–4]. Moreover, numerous studies suggest that the modification of their expression levels, sequence, or structure, has a relevant impact in many human diseases, including cancer [1–3]. Commonly, most *PRDM* genes express two main molecular variants, with (PR + product) and without (PR − product) the PR/SET domain, playing opposite roles, with the full-length product usually acting as a tumor suppressor, and the short one as an oncogene. The imbalance in their expression levels is observed in many cancers because of inactivating mutations or silencing of the PR + product and/or to increased expression of the PR − one [1–3]. Likewise, *PRDM2/RIZ* expresses two main molecular variants, the PR + isoform (*PRDM2a/RIZ1*) and the PR − (*PRDM2c/RIZ2*) [1–3, 5]. The imbalance in their expression levels in favor of *RIZ2* is observed in many cancer types. Formerly, the full length *RIZ1* was extensively investigated in several cancers where it acts as a tumor suppressor, whereas few studies had explored the oncogenic properties of *RIZ2*. Recently, we showed that *RIZ2* overexpression increased cell viability and growth, prompted the G2-to-M phase transition and organoids formation in HEK293 cells [5, 6]. Consistently, our Exome- and RNA-Seq public datasets available at The Cancer Genome Atlas (TCGA) portal analysis revealed that a subset of *PRDMs*, including *PRDM2*, are frequently mutated and/or transcriptionally deregulated in certain tumor types, such as colorectal cancer (CRC) [5, 7]. In addition, *PRDM2* is often target of frameshift mutations and aberrant DNA methylation in CRC [5, 8–10]. Particularly, frameshift mutations in the C-terminal region of *PRDM2*, affecting (A)8 or (A)9 repeats within exon 8, are found in one third of CRC with microsatellite instability [11–13]. These frameshift deletion mutations, enriched in CRC exhibiting microsatellite instability (MSI), rise to a truncated protein lacking the C-terminal PR-binding motif that is essential for the methyltransferase activity of PR/SET domain [14].

CRC is the third most deadly and fourth most diagnosed cancer worldwide. Despite the progress in early diagnosis and therapeutic options, CRC shows a poor prognosis with a 5 year survival rate of ~ 45%. The CRC first-line treatment involves a multimodal approach that usually comprises surgical resection and chemotherapy combined with monoclonal antibodies or proteins against vascular endothelial growth factor and epidermal growth factor receptor (EGFR) [15]. Nevertheless, CRC often relapses. As such, new efforts are needed to improve the screening, the therapeutic options and outcomes in CRC patients. In this context, the discovery of new druggable biomarkers and targets is still a challenge in clinical management of CRC patients.

We recently provided novel insights on the RIZ2 tumor-promoting functions in the HEK-293 cell model, highlighting its putative mechanism in cancer initiation and progression [6]. However, many relevant aspects of PRDM2 action in cancerogenesis remain to be elucidated, particularly those related to RIZ2 role in CRC pathogenesis. The present study aims to fulfill this gap and elucidate the tumor-promoting function of RIZ2 in CRC. To this aim, the biological outcomes of RIZ2 overexpression have been explored, using functional and transcriptome studies in CRC-derived DLD1 cells.

## Methods

### In silico analysis of RIZ1 and RIZ2 expression in colon cancer

Gene Expression Profiling and Interactive Analyses2 (GEPIA2) (http://gepia2.cancer-pku.cn/#index) [16] is an online resource for transcriptional profiles analysis, containing 275 colon adenocarcinoma (COAD) and 41 colon normal samples of “The Cancer Genome Atlas” (TCGA) [17]. We performed a differential analysis of *RIZ1* and *RIZ2* expression through GEPIA2 and used a boxplot to illustrate the results with log_2_ of transcript count per million [log_2_(TPM + 1)] showing the expression level of both isoforms. We also provided the expression distribution of *RIZ1*and *RIZ2* signatures in the 3 COAD subtypes.

### RNA extraction, quantitative reverse transcriptase polymerase chain reaction (RT-PCR)

Total RNAs were extracted from cells using Trizol solution (Thermofisher), according to the manufacturer’s instructions. The quality and quantity of RNAs were assessed by denaturing agarose gel electrophoresis and by spectrophotometry analysis (NanoDrop Technologies), after RNAse-free DNAse-I treatment (Boehringer Mannheim, Indianapolis, IN, USA). RNA was reverse transcribed with SuperScript III (Thermofisher) using 500 ng of total RNA. Quantitative RT-PCR analysis was performed as previously reported [4]. Glyceraldehydes-3-phophate dehydrogenase (*GAPDH*) or peptidylprolyl isomerase A (*PPIA*) were used as housekeeping control genes [18].

### Cell culture and treatments

Human colorectal adenocarcinoma cell lines DLD1, HCT116, SW48, SW620 and HT29 were provided and grown as previously described [19, 20].

When indicated 48 h before stimulation, 70% confluent growing cells were made quiescent using phenol red-free RPMI medium containing 0.1% charcoal-stripped serum (CSS), penicillin (100 U/ml) and streptomycin (100 U/ml).

The hEGF (Sigma Aldrich, St. Louis, Missouri, USA) was used at 100 ng/ml. The EGFR tyrosine kinase inhibitor, ZD1839, (Selleckem, Planegg, Germany) was used at 2 μM. The EGF neutralizing antibody (10605-R001; Sino Biological, Beijing, China) was used at 1.5 µg/ml.

### Plasmids and DLD1 transfection

The pEGFP-C1 vector was purchased from Clontech (Palo Alto, CA, USA) and was used to clone in the BamH1 site, the sequence of RIZ2 open reading frame (NM_015866.4) as reported elsewhere [4, 6]. The RIZ2 expressing plasmid, designated pEGFP_hRIZ2, was in frame with the EGFP coding sequence, which was positioned at the N-terminus, without in-frame stop codons. Based on previous observations, the produced fusion protein is likely to maintain the native RIZ2 functions [4–6]. Plasmids were prepared with Plasmid Midi Kit (Qiagen Inc, Valencia, CA, USA), according to manufacturer’s instructions. Cells were transfected using Lipofectamine^™^ 2000 Reagent in OptiMem I Reduced Serum Medium (Life Technologies, Carlsbad, CA, USA) for 6 h, following the manufacturer’s instructions. Stable clones were selected with 0.8 mg/ml Geneticin- G418 (Sigma-Aldrich). Transfection was verified by fluorescent microscopy and Western blot analyses [4, 6].

### Transcriptome profiling and bioinformatic analyses

Total RNA–seq procedure was performed as described previously [21, 22]. Briefly, before use, total RNAs concentration, from three biological replicates of DLD1-pEGFP and DLD1-pEGFP_hRIZ2 cells, was measured using RNA HS kit on a Qubit fluorimeter (Life Technologies, Monza, Italy) while its quality and integrity assessed with the Agilent 4200 TapeStation System (Agilent Technologies, Milan, Italy). For RNA sequencing experiments, indexed libraries, from the biological replicates mentioned above, were prepared using 500 ng of total RNA as starting material using the Illumina Stranded Total RNA prep Ligation with Ribo-Zero Plus kit (Illumina Inc., San Diego, CA, USA). Final libraries were pooled and diluted to a final concentration of 1.4pMol and sequenced on NextSeq 500 (Illumina Inc) in a paired-end mode (2 × 75 base pairs). The raw sequence files generated (.fastq files) underwent quality control analysis using FASTQC (http://www.bioinformatics.babraham.ac.uk/projects/fastqc/) and adapter sequences were removed using Trimmomatic version 0.38 [23]. Filtered reads alignment on the human reference genome (GRCh38/hg38) using STAR v2.7.5a with standard parameters [24]. Quantification of expressed genes was obtained with featureCounts [25]. Differentially expressed RNAs were identified using DESeq2 [26]. RNA expression was assessed when detected by at least ≥ 10 raw reads. Differential expression was reported as |fold change| (FC) ≥ 1.5 along with associated adjusted p-value or p-value ≤ 0.05 computed according to Benjamini–Hochberg as previously described [27]. Functional annotation analyses of differentially expressed genes were performed according to IPA (Ingenuity Pathway Analysis, QIAGEN) [28] and GEPIA2 [16] that was used also for gene expression correlation analyses.

### Immunofluorescence, DNA synthesis, cell number and WST-1 assay

DLD1-pEGFP and DLD1-pEGFP_hRIZ2 were analyzed for pEGFP expression by immunofluorescence microscopy as reported [6].

Cells plated on gelatin-coated coverslips were made quiescent and after 72 h were rinsed with phosphate-buffered saline (PBS), fixed for 20 min with paraformaldehyde (4%, w/v, in PBS; Merck, Saint Louis, MO, United States), permeabilized for 10 min with Tween (0.1%, v/v, in PBS; Bio-Rad, Hercules, CA, United States), and incubated for 1 h with PBS containing FBS (1%, vol/vol). Cells were then incubated with the anti-EGF (1:100, ab131498 Abcam, Cambridge, United Kingdom) antibody for 2 h. After washings in PBS, the coverslips were incubated for 1 h at 37 °C with diluted (1:250 in PBS, containing 0.01% BSA) Texas red-conjugated AffiniPure anti-rabbit immunoglobulin G (IgG; Jackson ImmunoResearch Laboratories, West Grove, PA, United States). Nuclei were stained for 5 min with Hoechst 33258 (1 μg/ml; Merck). The number of EGF-positive cells was determined as percentage of EGF-positive cells on total cells.

The DNA synthesis was analyzed by BrdU incorporation. Cells were left unchallenged or challenged as indicated in the figures and in corresponding legends. After in vivo pulse with 100 μM BrdU (Sigma-Aldrich, St. Louis, MO, United States), BrdU incorporation into the newly synthesized DNA was analyzed and quantified [29], using a DMLB (Leica, Wetzlar, Germany) fluorescent microscope equipped with HCX PL Apo × 63 oil and HCX PL Fluotar × 100 oil objectives. Images were captured using a DC480 camera (Leica) and acquired using the Leica Suite software.

DLD1-pEGFP and DLD1-pEGFP_hRIZ2 cells were counted in the Bürker chamber by optical microscopy. DLD1-pEGFP_hRIZ2 cells were compared with DLD1-pEGFP cells.

Cell proliferation was determined using the WST-1 reagent (Merck). Untreated or treated 1 × 10^3^ cells in 96-well culture plates were incubated with the WST-1 reagent for 2.5 h. Thereafter, the formazan dye was quantified at 450 nm by a scanning multi-well spectrophotometer (Enspire; Perkinelmer, Waltham, Massachuttes, USA). The measured absorbance was correlated to the number of viable cells.

### Clonogenic assay

DLD1-pEGFP and DLD1-pEGFP_hRIZ2 cells (1.5 and 3 × 10^2^) were seeded into six-well plates and cultured at 37 °C for ~ 10 days until cells have formed sufficiently large clones (at least 50 cells). Fresh media were supplied every 3 days. Clones were counted after 30 min fixing with a mixture of 6% glutaraldehyde and 0.5% crystal violet [6, 30]. The stained colonies were photographed and the number colonies with size ≥ 1 mm were counted using the ImageJ software (National Institutes of Health, USA) and expressed as mean ± S.E.M. Each assay was performed in at least three independent experiments in triplicate.

### Wound scratch analysis, migration, invasiveness and phase-contrast microscopy

For wound scratch analysis, 1.5 × 10^5^ cells were seeded in a 24-well plate and wounded with a 10 µl sterile pipette tip. Cells were washed with PBS and pre-incubated for 30 min with cytosine arabinoside (Sigma-Aldrich) at 50 µM (final concentration) to inhibit cell proliferation. Cells were then left untreated or treated, as indicated in the figures. Different fields were analyzed using DMIRB inverted microscope (Leica) equipped with N-Plan 10 × objective (Leica) [31]. Phase-contrast images were captured using a DFC 450C camera (Leica) and acquired using Application Suite Software (Leica). Images are representative of at least three different experiments. The wound area was calculated using NIH Image J Software and expressed as % of residual area.

The migration and invasion assays were done using 2.5 × 10^4^ cells in Boyden’s chambers with 8 μm polycarbonate membrane (Corning; Corning, NY, USA) pre-coated with Collagen or growth factor reduced and phenol red-free Matrigel (Corning; Corning, NY, USA), respectively. Cytosine arabinoside (at 50 µM) was included in the cell medium. After 7- or 18 h respectively, non-migrating or non-invading cells were removed from the upper surface membrane using a sterile cotton swab. Membranes were fixed for 20 min in 4% paraformaldehyde, stained with Hoechst, removed with a scalpel from the companion plate and mounted. Migrating or invading cells from at least 30 fields/each membrane were counted as described [32]. Data are representative of at least three independent experiments.

### 3D cultures

Organoids were generated as reported [31]. Cells (1.5 × 10^4^) were mixed in each well with 250 μl of growth factor reduced and phenol red-free Matrigel (Corning) and 100 μl of organoid plating medium (DMEM/F12 medium, containing 5%FBS, penicillin (100U/ml), streptomycin (100 U/ml), diluted GlutaMAX (100X), 10 mM Hepes, B27 (50 × stock solution), 1 mM nicotinamide (Merck), 1.25 mM N-acetylcysteine (Sigma-Aldrich), and 10 μM Y-27632 (Merck-Millipore, Temecula, CA, USA). After 3 days, the organoid-plating medium was replaced with a similar medium without N-acetylcysteine and Y-27632. When indicated, the organoids were untreated or treated with the indicated compounds. Except for the experiments with the EGF neutralizing antibody [29], the medium was changed every 3 days and different fields were analyzed using DMIRB Leica (Leica) microscope, equipped with C-Plan × 40 (Leica). Phase-contrast images were acquired using a DFC 450C camera (Leica). The relative organoid size was calculated using the same software and expressed as a fold increase over the basal organoid size.

### Enzyme-linked immunosorbent assay

Cells (1 × 10^6^) were seeded in 100 mm plates and made quiescent. Cell culture media were collected after 24-, 48- and 72 h and used to assay the EGF concentration, according to the manufacturer’s instructions. Human EGF (KHG0061; Invitrogen) ELISA kit was used. Data were analyzed using the curve-fitting statistical software Graph Prims Pad.

### Lysates and western blot

Lysates were done as reported [31]. The following reagents were used: anti-KMT8/Riz1/Riz2 antibody (ab3790; Abcam); mouse monoclonal anti-FAK (610,088; BD Transduction Laboratories), or anti P-Tyr 397 FAK (611722; BD Transduction Laboratories); anti-p42 extracellular signal-regulated kinase (ERK) (sc-1647; Santa Cruz), or anti p44 and p42 P-ERK (sc-7383; Santa Cruz); anti-tubulin (DM1A sc-32293; Santa Cruz), anti-GAPDH (#E-AB-20078; Elabscience) and rabbit polyclonal antibodies anti-EGFR (610016;Millipore), anti P-Tyr1068 EGFR (#2234S; Cell Signaling, Danvers, MA, United States) antibodies. The ECL system (GE Healthcare, Chicago, IL, United States) was used to reveal immunoreactive proteins.

### Statistical analysis

Results from cellular and biochemical data are reported as mean ± SD. Three independent experiments in triplicates were performed. GraphPad Prism 9,5 software was utilized to perform Brown-Forsythe and Welch ANOVA tests. Significances are indicated in the corresponding legends.

## Results

### In silico analysis of TCGA-COAD dataset revealed a PRDM2 isoform imbalance vs RIZ2.

We first evaluated the expression of the different *PRDM2* transcripts by in silico analysis of TCGA-COAD (colon adenocarcinoma) datasets on GEPIA2 (Additional file 2: Fig S1) [16, 17]. Based on the Isoform Usage, a highly significant *RIZ2* expression *vs RIZ1* transcript was observed in violin plots from COAD samples (Additional file 2: Fig S1A). Additionally, for the isoform analysis in box plot, *RIZ2* was overexpressed in tumor samples compared to normal tissues whereas *RIZ1* was downregulated even though not significantly (Additional file 2: Fig S1B). Interestingly, the *RIZ2* overexpression was higher in colon cancers with Microsatellite stability (MSS) (Additional file 2: Fig S1C). This expression pattern suggested a putative role of *RIZ2* as an oncogene in colon cancer.

### PRDM2 transcript levels in CRC cell lines and RIZ2 ectopic overexpression in DLD1 cells

We performed a *PRDM2* expression analysis by qRT-PCR in several CRC cell lines with different microsatellite instability (MSI) status (DLD1, HCT116, HT29, SW48 and SW620) [33]. *PRDM2* expression was verified using two sets of primers: 118F/438R recognizing sequences on exons 3 and 5 (*RIZ1 specific*) and 649F/975R on exon 8 (common to both *RIZ1* and *RIZ2* and indicated as *RIZex8*). Because of the extensive similarity between the two gene products, *RIZ2* transcript was measured by subtraction as previously described [4, 34]. Interestingly, we found that the only cell line with MSS (SW620) expressed higher RIZ2 transcript levels compared to the others with MSI (DLD1, HCT116 and SW48) (Additional file 2: Fig S1D) thus paralleling the in-silico data on TCGA-COAD patients (Additional file 2: Fig S1C). This analysis revealed a lower expression of *PRDM2* transcripts in DLD1 cells than other CRC cell lines analyzed (Additional file 2: Fig S1D).

**Fig. 1:**
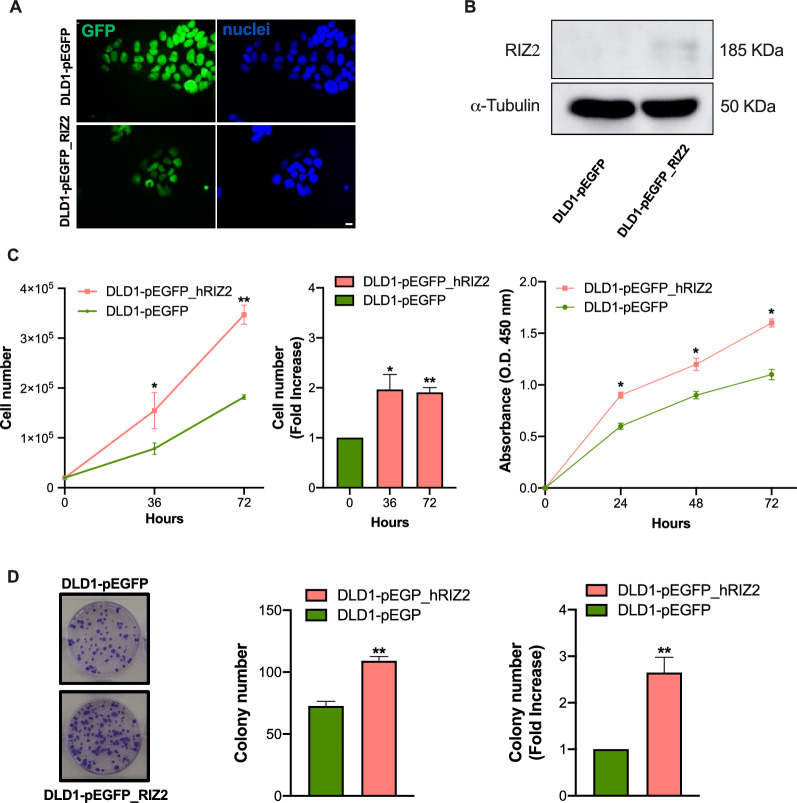
RIZ2 overexpression in DLD1 cell line. **A** Immunofluorescence analysis of GFP in DLD1-pEGFP and DLD1-pEGFP_hRIZ2. Nuclei are stained in blue. Images were captured using a DC480 camera (Leica) and acquired by Application Suite (Leica) software and are representative of three independent experiments. **B** Western blot analysis with an antibody that recognizes both RIZ2 (185 kDa) and RIZ1 proteins. **C** Effects of *RIZ2* overexpression on DLD1 cell viability. Cell number was obtained by microscopy count or extrapolated by WST-1 assay. DLD1 cells stable transfected with pEGFP_hRIZ2 or with pEGFP alone, as control, were cultured for 36- and 72 h and counted in the Bürker chamber. Fold increase *vs* the control cells is also illustrated. DLD1 cells stable transfected with pEGFP_hRIZ2 or with pEGFP alone, as control, were cultured for 24-, 48-, and 72 h and assayed with WST-1. Graphs represent the extrapolated cell number at the different time points. Three independent experiments were performed. *p < 0.05 and **p < 0.0001 for the indicated experimental points vs. the corresponding untreated control. **D** Representative images of clonogenic assay. Ten days after seeding, clones were counted, and their cellularity was evaluated by phase contrast microscopy. The histogram represents the average number of colonies of at least three independent experiments, each performed in triplicate (**p < 0.0001 vs. empty vector); on the right the fold change in colony number formation between pEGFP_hRIZ2 cells and pEGFP control cells (**p < 0.0001 vs. empty vector)

To assess the *PRDM2/RIZ2* role in CRC cell behavior, DLD1 cells were stably transfected with a plasmid encoding for *RIZ2* in frame with E-GFP (pEGFP_hRIZ2) and with the E-GFP empty vector (pEGFP). Thus, the balance between the isoforms was modified in favor of *RIZ2*, reproducing a condition often observed in cancer and in our *in-silico* analysis [6, 7]. The RIZ2 overexpression was verified after transfection by fluorescence microscopy and Western blot analysis (Fig. 1A, B).

### RIZ2 Overexpression in DLD1 colon cancer cells prompts cell viability, motility and 3D-organoid growth

To investigate the possible oncogenic properties of *RIZ2* in CRC, we analyzed stable transfected DLD1 cells with pEGFP_hRIZ2 or with pEGFP control vector through several functional assays, including cell growth, colony formation, motility and organoid formation (Fig. 1). Cell viability and proliferation analysis through cell count and WST-1 assay showed that the number of DLD1-pEGFP_hRIZ2 cells at 24-, 48- and 72 h was significantly higher than DLD1-pEGFP control cells (Fig. 1C). Specifically, *RIZ2* overexpressing cells exhibited about two folds increase in cell viability than control cells at 48- and 72 h. To deeper assess the role of RIZ2 in tumorigenesis, we analyzed the ability of DLD1 stable clones overexpressing pEGFP_hRIZ2 or the pEGFP control vector to form colonies. *RIZ2* overexpression induced the formation of a markedly higher number of colonies than the control cells (*P < 0.001 *vs* empty vector) (Fig. 1D). Specifically, DLD1-pEGFP_hRIZ2 cells showed about a two folds increase in the number of colonies.

**Fig. 2:**
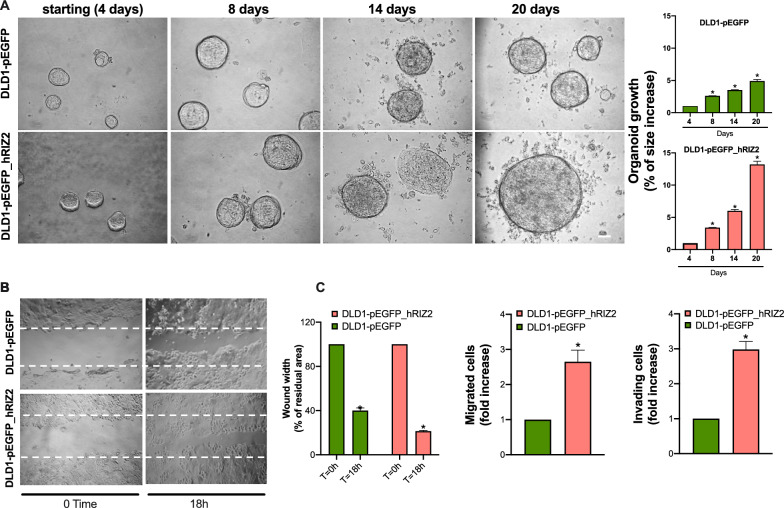
RIZ2 overexpression Increases DLD1 3D-organoid growth and cell migration. **A** Representative images of stable transfected DLD1 cells in miniaturized 3D cultures in extracellular matrix (three independent experiments, each performed in duplicate). Scale bar, 100µ. Graphs illustrate the relative organoid size (area), which was calculated using the Application Suite Software and expressed as a relative fold increase at 8, 14 and 20 days over the organoid area calculated at 4th day. *p < 0.05 *vs.* control cells. **B** DLD1-pEGFP cells and DLD1-pEGFP_hRIZ2 were wounded and left for 18 h. Phase-contrast images are representative of three different experiments, each in duplicate. **C** The wound area was calculated using Leica Suite Software. Data are represented as % of residual wound width. The standard deviations were < 0.05 for each experimental condition (left graph). In middle and right graphs, DLD1-pEGFP cells and DLD1-pEGFP_hRIZ2 were allowed to migrate (middle) or invade (right) for 7 h or 24 h, respectively. They were scored as described in “Methods” section and data were expressed as fold increase. Means and SDs are shown. n = 3. *p < 0.05 for the indicated experimental points versus the corresponding 0 time cells

To deeper investigate the biological effects elicited by *RIZ2* overexpression in colon cancer, 3D organoids were established, as they resume better the cancer tissues characteristics reproducing the complex in *vivo* architecture and represent a powerful tool for evaluating the efficacy of drug treatment. Phase-contrast images at 4 days revealed a 3D structure in both DLD1-pEGFP_hRIZ2 and DLD1-pEGFP control cells cultured in Matrigel. Both cell types generated roundish and well-differentiated organoids. Changes in dimension and structure of organoids were then monitored for additional 16 days. Phase-contrast microscopy images at 8th, 14th and 20th day were captured and shown. Data quantification showed that DLD1-pEGFP organoid size was significantly (p < 0.05) increased by about 2.6- and 3.5- and 4.9-fold after 8, 14 and 20 days, respectively. Notably, we observed a higher and significant (p < 0.05) increase (about 3.4-, 6- and 13.2-fold at 8, 14 and 20 days, respectively) in the size of DLD1-pEGFP_hRIZ2-derived organoids (Fig. 2A). These findings demonstrate a role for *RIZ2* in the growth of DLD1-derived organoids. Overall, these results confirm our previously findings in HEK293 cells, demonstrating the *RIZ2* tumor promoting properties [6].

In this cellular model, we also evaluated the effect of *RIZ2* on migration and invasiveness. To this purpose, DLD1 cell lines were wounded and allowed to migrate. Phase-contrast images from wound scratch assay revealed that DLD1-pEGFP_hRIZ2 cells had a significantly higher migration rate than control cells (Fig. 2B). Data from three independent experiments indicated that the wound width was significantly (p < 0.05) reduced in DLD1-pEGFP_hRIZ2 cells, as compared with the DLD1-pEGFP control cells. Furthermore, *RIZ2* overexpression significantly increased the number of migrating and invading cells (2.65- and 2.98-fold, respectively) (Fig. 2C).

### RIZ2 overexpression impact transcriptome profile of DLD1 cells via EGF signaling pathway deregulation

**Fig. 3:**
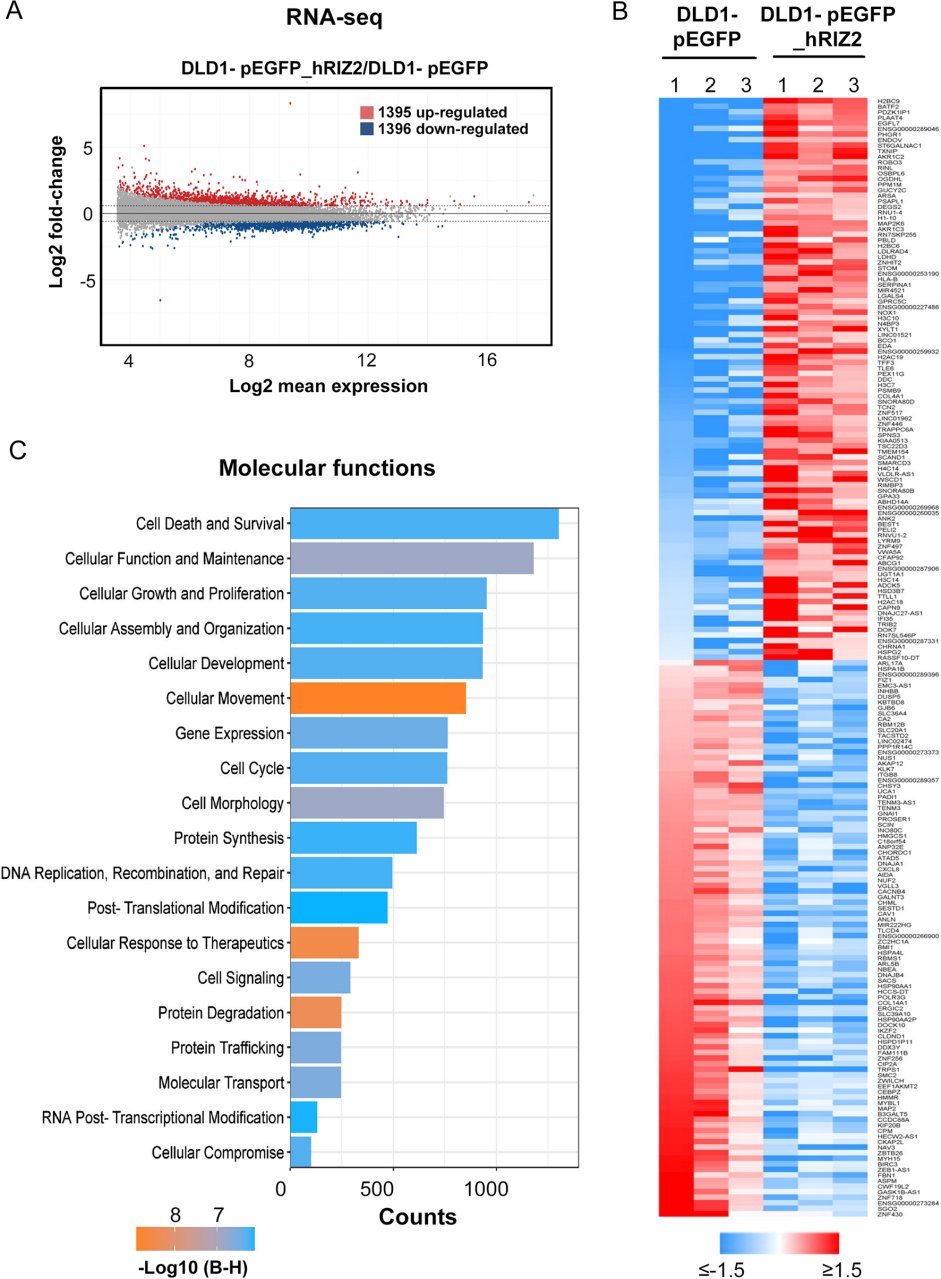
Impact of RIZ2 overexpression on gene expression in DLD1 CRC cells. **A** MA plot showing transcriptome changes, measured by RNA sequencing (RNA-seq), following *RIZ2* overexpression. **B** Heatmap showing top 100 down-regulated (blue) and up-regulated (red) transcripts following *RIZ2* overexpression. (fold-change ≤ and ≥|1.5|, adj p-value < 0.05). Data are shown as normalized expression values in log2 scale and centered on the median value. **C** Bar Chart obtained by IPA (Ingenuity Pathway Analysis) functional annotation analysis showing statistically significant molecular function enriched following *RIZ2* overexpression

**Fig. 4:**
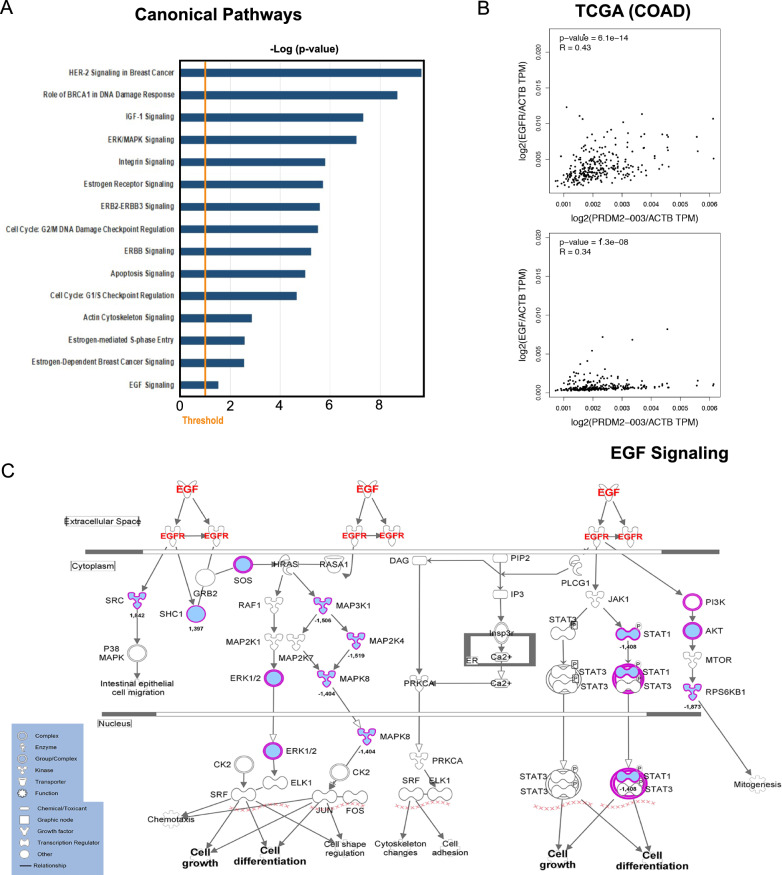
RIZ2 overexpression deregulate EGF-R pathway in DLD1 CRC cells. **A** Bar Chart obtained by IPA (Ingenuity Pathway Analysis) functional annotation analysis showing statistically. Significant pathway enriched following *RIZ2* overexpression. **B** Results of TCGA data analysis. Correlation of *RIZ2* (PRDM2-003) and *EGFR* or *EGF* mRNA expression in COAD tumor samples obtained by GEPIA2. **C** Ingenuity Pathway Analysis of the EGF/R (red) pathway highlighting changes in transcripts (blue) induced by *RIZ2* overexpression

To investigate the functional role of RIZ2 in CRC, exponentially growing DLD1-pEGFP_hRIZ2 cells and DLD1-pEGFP control cells were gene expression profiled. As shown in Fig. 3, *RIZ2* overexpression determined a profound effect on DLD1 cell transcriptome, including 2788 transcripts affected (1396 down- and 1395 up-regulated, compared to control cells; fold-change ≤ and ≥|1.5|) (Fig. 3A, Additional file 1: Table S1) with the top 100 up- and down- regulated ones depicted in the in the heatmap shown in Fig. 3B. Transcriptome changes induced by *RIZ2* overexpression highlighted an effect on crucial molecular function, such cell death and survival, cell growth and proliferation, cellular movement and cell cycle mediated by changes in several deregulated genes known to control several key features of CRC cells (Fig. 3C). Thus, results obtained at transcriptome level confirmed the DLD1-pEGFP_hRIZ2 behavior observed by cellular and molecular assays described above. Transcriptome data analysis by IPA strengthen and expanded these results, showing a significant effect on a series of signaling pathways including Apoptosis and Cell Cycle G1/S regulation [35, 36] but also and more interestingly ERK-MAPK, Estrogen and EGF signaling (Fig. 4A). In particular, the EGF/EGFR pathway caught our attention since EGFR has been shown to be overexpressed in colorectal cancer patient populations but its prognostic value in this tumor progression remains still unclear [35, 36]. Thus, we employed TCGA dataset analysis via GEPIA2 to correlate *PRDM2/RIZ2* with *EGF* and *EGFR* expression levels in CRCs specimen’s cohort. The results highlighted a correlation between *RIZ2* and both *EGF* and *EGFR* expression levels (Fig. 4B) and an extensive modulation of several key nodes of the aforementioned pathway leading to cell growth and differentiation (Fig. 4C).

### The EGFR tyrosine kinase inhibitor ZD1839 greatly neutralized RIZ2 oncogenic properties

**Fig. 5:**
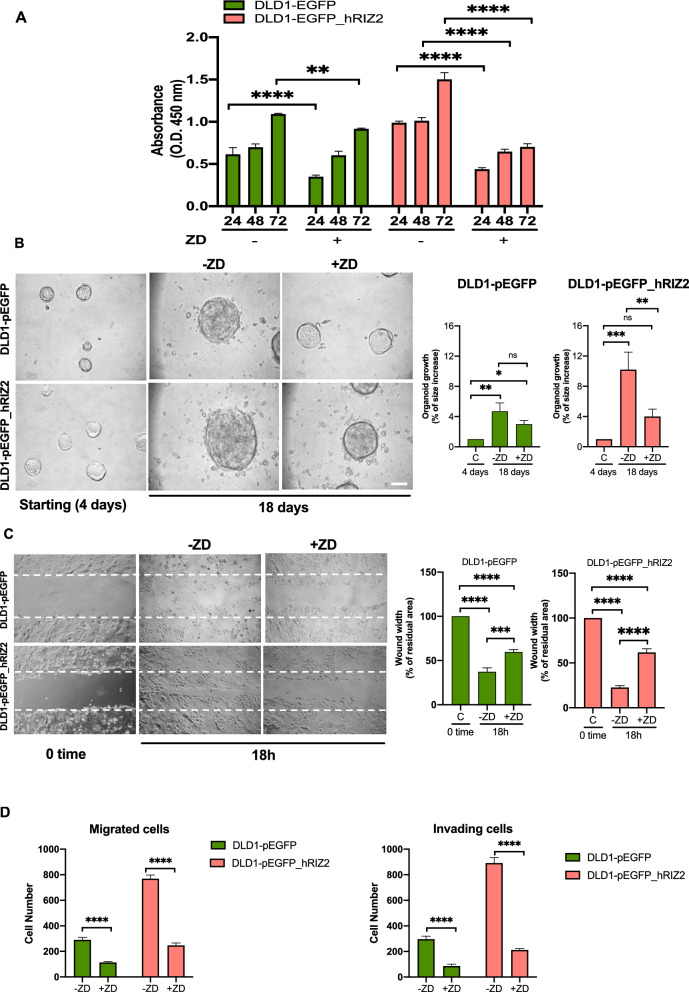
ZD1839 greatly neutralized RIZ2 oncogenic properties. In **A**–**D**, DLD1-pEGFP and DLD1-pEGFP_hRIZ2 stable cell lines were used. Cells were untreated or treated with ZD1839 (ZD) and analyzed for cell proliferation (**A**) through WST-1. **B** Cell lines were used in miniaturized 3D cultures in extracellular matrix (ECM), as reported in Methods. Four days after cells embedding in Matrigel (starting) representative images were acquired as described in Methods and cells were unchallenged or challenged with ZD1839 (ZD) for 18 days. Shown are phase-contrast images captured on the 18th day. Scale bar, 100 µ. Graphs on the right show the analysis of organoid size from cell lines unchallenged or challenged with ZD. The size of organoids was calculated using Leica suite software. For each cell line, 3 different experiments were done. Means and SDs are shown. Cell migration was analyzed through wound scratch assay (**C**) or collagen-coated transwells (**D**, left panel) and cell invasiveness trough Matrigel-coated transwells (**D**, right panel) as described in Figs. 1, 2 legends and Methods section. In **A**–**D** means and SDs are shown. *p < 0.05; **p < 0.001; ***p < 0.0005; ****p < 0.0001; *ns* not significant

Based on our transcriptomic results, we investigated whether the imbalance of *RIZ* isoforms in CRC cells could affect the EGFR signaling pathway, by targeting it through the selective tyrosine kinase inhibitor ZD1839. Thus, DLD1-pEGFP and DLD1-pEGFP_hRIZ2 stable cells were treated with ZD1839 and analyzed for their oncogenic properties (Fig. 5). WST-1 assay showed that ZD1839 was able to significantly counteract the effect of *RIZ2* overexpression on cell survival starting from 24 h and this inhibition was maintained at 48 h and 72 h (Fig. 5A). In DLD1-pEGFP cells, a significant inhibition of the proliferative rate was observable at a lesser extent only at 24 h, whereas a minimal non-significant effect on proliferation was revealed at the subsequent time points (Fig. 5A). Similarly, ZD1839 significantly interfered with spheroid growth measured as a fold increase of the organoid area after 18 days from the starting point (4th day). A higher reduction was observed in DLD1-pEGFP_hRIZ2 cells (4 *vs* 10.2) compared with DLD1-pEGFP cells (3.3 *vs* 4.7) (Fig. 5B). Likewise, we evaluated migration and invasiveness (after ZD1839 treatment of DLD1-pEGFP and DLD1-pEGFP_hRIZ2 stable cells with different approaches (Fig. 5C, D). Interestingly, the inhibition of EGFR phosphorylation prevented cell migration (Fig. 5C, D, left panel) and invading capacities of DLD1-pEGFP_hRIZ2 cells whereas a small but significant effect was observed in DLD1-pEGFP cells (Fig. 5D).

### RIZ2 overexpressing cells release high EGF levels thus activating an autocrine regulation of CRC cell behavior

**Fig. 6:**
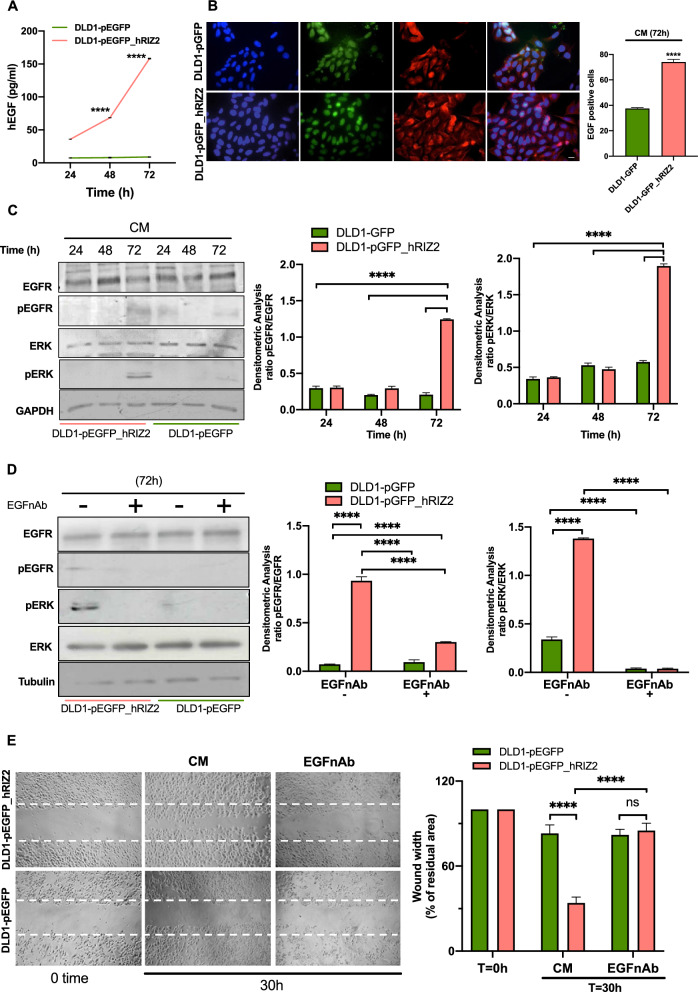
*RIZ2* overexpressing cells release high EGF levels. **A** EGF concentration in cell culture medium was measured through ELISA method at different time points (24-, 48- and 72 h) in quiescent DLD1-pEGFP and DLD1-pEGFP_hRIZ2. **B** Immunofluorescence analysis with the anti-EGF antibody (red) on DLD1-pEGFP and DLD1-pEGFP_hRIZ2 positive cells (green). Nuclei are stained in blue. Scale bar, 5 µm. **C** DLD1-pEGFP and DLD1-pEGFP_hRIZ2 transfected cells were left unchallenged for (24-, 48- and 72 h), avoiding refreshing the medium. EGFR and ERK phosphorylation were revealed by Western blot and densitometry analysis. **D** DLD1-pEGFP and DLD1-pEGFP_hRIZ2 transfected cells were left untreated for 72 h, avoiding refreshing the medium, in the absence or presence of the anti-EGF neutralizing antibody (anti-EGF nAb). Analysis of EGFR and ERK phosphorylation after treatment with anti-EGF nAb. **E** DLD1-pEGFP and DLD1-pEGFP_hRIZ2 transfected cells were wounded and left untreated for 30 h avoiding refreshing the medium, in the absence or presence of the anti-EGF antibody (anti-EGF Ab) for analyzing the cell migration induced by conditioned medium. Wound area was calculated using Leica Suite software. Data are presented as the percentage of residual area in wound width over the control cells, analyzed at time 0. Means and SDs are shown. Pictures are representative of three different experiments. ****p < 0.0001; ns not significant

At last, we hypothesized that *RIZ2* overexpressing cells release EGF in the culture medium. Thus, we assayed EGF concentration in cell culture media collected at different time points (24-, 48- and 72 h) in quiescent DLD1-pEGFP and DLD1-pEGFP_hRIZ2 cells. At all time points we detected significantly higher EGF levels in DLD1-pEGFP_hRIZ2 cells, as compared with DLD1-pEGFP cells (Fig. 6A). Indeed, *RIZ2* overexpressing cells exhibited about 35-, 68- and 158 pg/ml EGF at 24-, 48- and 72 h respectively. By contrast, medium from control cells contained about 7–8 pg/ml EGF at every time point.

Interestingly, this finding was also corroborated by further data. Indeed, immunofluorescence quantification and images (Fig. 6B) reveal that a significant number of DLD1-pEGFP_hRIZ2 cells were positive for EGF immunostaining, as compared with control cells (74% *vs* 37.5%). In both cell lines, EGF staining was prevalently seen in the extranuclear compartment. The absence of fluorescence in cells stained with the secondary antibody alone indicates the specificity of our IF approach (Additional file 2: Fig S2).

Thus, we argued that several downstream effectors of the EGF/EGFR axis are activated by the EGF-mediated autocrine loop. The Western blot and densitometry analysis (Fig. 6C**)** show that conditioned medium (CM) promoted the phosphorylation of EGFR at 72 h in DLD1-pEGFP_hRIZ2 cells, without affecting the total protein levels. This is an important step, since the EGFR phosphorylation and, thus, its activation is responsible of the consequent activation of ERK. This pathway triggers cellular responses such as proliferation, migration and invasiveness. The addition of neutralizing anti-EGF antibody (EGFnAb) prevented the EGF-induced activation of EGFR and ERK (Fig. 6D).

The EGF-mediated autocrine loop also triggers cell motility, as wound scratch assay reveals that CM greatly increased the basal DLD1-pEGFP_hRIZ2 cell migration rate than DLD1-pEGFP control cells (wound width 34 and 83% respectively) (Fig. 6E). Treatment with EGFnAb consistently reversed the CM effect on cell migration (Fig. 6E).

**Fig. 7:**
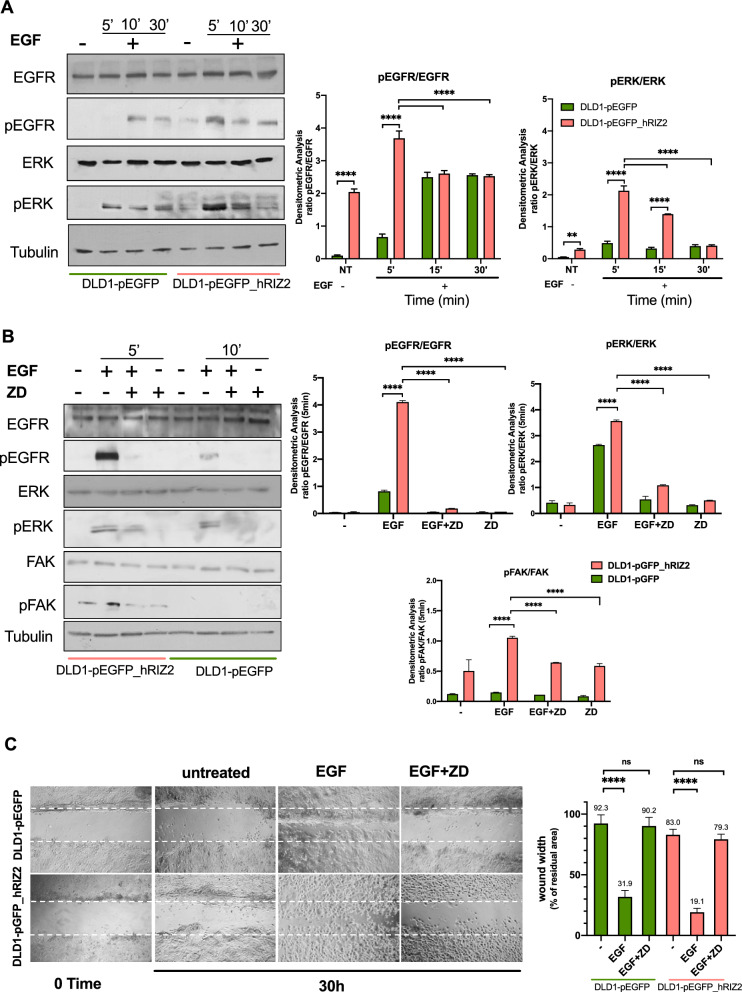
EGF stimulation of DLD1 transfected cells. **A** Quiescent DLD1-pEGFP and DLD1-pEGFP_hRIZ2 were untreated or treated with exogenic hEGF at short time points (5-, 10- and 30 min). EGFR and ERK were analyzed by Western blots, which are representative of three different experiments. Expression levels of proteins were analyzed by densitometry analysis, using NIH Image J Software. The ratio between pEGFR/EGFR (left graph) and pERK/ERK (right graph was evaluated. Results were expressed as relative ratio. Means and SDs are shown. **B** The effect of ZD1839 on EGFR and ERK phosphorylation in EGF stimulated cells at 5 min (DLD1-pEGFP_hRIZ2 cells) and 10 min (DLD1-pEGFP cells). Phosphorylation of FAK was also evaluated. Densitometric analysis was performed as in A. **C** DLD1-pEGFP and DLD1-pEGFP_hRIZ2 quiescent cells were wounded and left untreated or treated for 30 h in absence or presence of EGF and ZD1839, as indicated in the figure. Wound area was calculated using Leica Suite software. Data are presented as the percentage of residual area in wound width over the control cells, analyzed at time 0. Means and SDs are shown. Pictures are representative of three different experiments. ****p < 0.0001; *ns* not significant

As readouts of EGF treatment, we analyzed the phosphorylated status of both EGFR and ERK. In a preliminary time-course experiment, quiescent DLD1 cell lines were challenged for different times (from 5 to 30 min) with 100 ng/ml EGF. A robust phosphorylation of EGFR and ERK was detectable within 5 min in DLD1 cells overexpressing *RIZ2* (right side). By contrast, a weaker activation was observed after 10 min of stimulation of the control cells. Furthermore, the kinetics of activation displayed small differences in the two cell lines (Fig. 7A).

Therefore, we selected these time points in the subsequent analysis. Quiescent DLD1 cells were then challenged with EGF for 5 (DLD1-pGFP_hRIZ2) or 10 (DLD1-pGFP) minutes respectively in the absence or presence of the selective tyrosine kinase inhibitor, ZD1839 [37]. Noteworthy, EGF-induced EGFR and ERK phosphorylation were counteracted by ZD1839 (Fig. 7B) in both cell lines. Besides, phosphorylation of focal adhesion kinase (FAK), a protein involved in the molecular machinery leading to cell locomotion [38], was found in DLD1-pEGFP_hRIZ2 cells, whereas it was undetectable in DLD1-pEGFP ones. EGF treatment fostered FAK activation, which was reversed by ZD1839 (Fig. 7B). Consistent with these data, the wound scratch assay gave similar results (Fig. 7C), indicating that the EGF-triggered FAK activation is involved in cell motility.

## Discussion

Human CRC is one of the most common types of malignancy and cancer-related death causes worldwide [39, 40]. Despite the progress in early diagnosis and treatment, CRC still shows a poor prognosis and a low survival rate also due to high incidence of recurrence and drug-resistance. Besides, the incidence rates, even among young generations, are expected to continuously increase especially in developing countries, thus implying the need of further efforts in the development of innovative tools for improved prevention and treatment of CRC. In this context, the discovery of new biomarkers and targets is a crucial goal for CRC management.

CRC is caused by genetic alterations that target oncogenes, tumor suppressors and DNA repair genes [41]. The pathogenic mechanisms include chromosomal instability (CIN), microsatellite instability (MSI) and CpG island methylator phenotype (CIMP), each of which is characterized by specific molecular profiles, stages of tumor development or progression, and, ultimately, prognosis. Moreover, also in the same genetic background, a peculiar feature of CRC is its heterogeneity, which explains the wide variability of response to systemic therapies characterized by some patients with satisfactory and sustained responses and other experiencing low sensitivity, relapse, rapidly progressive disease and poor prognosis. In the joint effort to define a classification able to stratify CRCs into distinct genetic subtypes and to program and optimize personalized first- and second-line chemotherapies for metastatic disease, the consensus molecular subtypes (CMS) have been described [42]. The CMS classify CRCs into four subtypes according to their molecular profiles and taking into account, beyond mutations, the anatomical location of the primary neoplasia and specific cellular subsets within the microenvironment able to influence malignant cells in various ways. However, the possibility to effectively translate the CMS into clinical practice and to select patient-tailored treatments is a matter of debate and reveals that several key players in CRC occurrence and progression, or at least several molecular interactions, are still under-recognized [43].

In CRC, common alterations affect crucial signaling pathways, as EGFR signaling pathway among the others [44, 45]. Indeed, the current CRC first-line treatment involves an approach that may comprise monoclonal antibodies or proteins against EGFR, combined with surgical resection and chemotherapy [15]. In this scenario, it is claimed to further deepen our understanding of CRC biology, spanning from gene expression control to signal transduction pathways and metabolic processes regulating CRC behavior.

Some *PRDMs* are mutated or aberrantly expressed in CRC [7]. Specifically, *PRDM2* gene is often target of aberrant DNA methylation and frameshift mutations in CRC; besides, most of the identified *PRDM2* mutations were enriched in cancers exhibiting MSI [1, 5, 8–11, 46, 47]. Furthermore, our *in-silico* analysis of transcript expression levels from TCGA-COAD dataset revealed an imbalance among *RIZ1* and *RIZ2* in favor of *RIZ2* in CRC (Additional file 2: Fig S1). Based on literature data and bioinformatics observations, as well as our previous findings in HEK293 model [6], we hypothesized that *RIZ2/RIZ1* imbalance could also play a role in CRC. Consistently, forced *RIZ2* overexpression in DLD1 colon cancer cells was able to increase cell growth, colony and organoid formation thus confirming its oncogenic function [6] (Figs. 1, 2). Additionally, DLD1 *RIZ2* overexpressing cells showed a higher ability to reduce the wounded areas in wound scratch assays and invade collagen or Matrigel thick layers more efficiently than DLD1 control cells, suggesting a role for RIZ isoform imbalance in regulating colon cancer cell migration and invasion (Fig. 2). Interestingly, in the present study we have further investigated the possible action mechanism by analyzing the sequenced transcriptome of *RIZ2* overexpressing cells [48]. As expected, these cells exhibited many differentially expressed genes (DEGs) involved in colon cancer progression and metastasis (Fig. 3). Noteworthy, among the enriched pathways, we noted the presence of EGFR signaling that might lead to malignant transformation and CRC progression [15, 44, 45] (Fig. 4). Thus, we hypothesized that activation of EGFR signaling represents a putative mechanism explaining the *RIZ2* oncogenic properties.

EGF ligands, including the EGF-related peptides, activate EGFR and other family members. Enhancement of EGFR ligand expression, an autocrine loop mediated by an EGFR ligand itself, is the main mechanism implicated in cancer development and progression [49–51]. CRC cells release EGF in tumor microenvironment [52]. EGF, in turn, can bind the EGFR expressed by surrounding monocytes leading to the activation of Smad-PI3K-Akt-mTOR pathway and the polarization of monocytes into M2 macrophages [52]. In such a way, colon cancer cells and tumor-associated macrophages are involved in a cross-talk that affects and enhances malignancy of colon cancer.

EGFR can be considered a biomarker of cancer aggressiveness, since metastatic cells express five times more EGFR than nonmetastatic cells [53]. EGFR is overexpressed in 60% to 80% of colon cancers, leading to development of EGFR-targeted drugs [54], an important tool for treating cancer. In addition, specific targeting of EGFR ligands (e.g. amphiregulin, heparin-binding EGF-like growth factor) by neutralizing antibodies or small molecules represents a promising therapeutic approach, as it inhibits the proliferation and metastatic events in breast [55] and hepatocellular [56] carcinoma cells.

Despite accumulating evidence suggests that the EGFR or transforming growth factor α (TGFα)/ EGFR signaling pathways play a critical role in colon cancer progression, the EGF autocrine loop in colon cancer is still poorly investigated. Targeting EGFR signaling through EGFR inhibitors profoundly impairs the tumor progression [57]; however, monoclonal antibodies blocking the ligand binding to EGFR, have shown modest activity as a single agent in patients with metastatic colorectal cancer in clinical trials. Thus, combinatorial approaches against still unexplored intracellular targets (e.g. EGFR and RIZ2 signaling network) would improve the current clinical management of CRC patients.

Here, we show that the blockade of EGFR phosphorylation by ZD1839 interferes with proliferation, spheroid size and invasion of *RIZ2* overexpressing cells (Fig. 5). These cells also release higher EGF levels, as compared to control ones suggesting the possibility that the EGF-mediated autocrine loop modulation triggered by RIZ2 contributes to the maintenance of aggressive cell phenotype (Fig. 6). In turn, conditioned medium promotes the phosphorylation of EGFR and ERK in DLD1 *RIZ2*-overexpressing cells. A neutralizing anti-EGF antibody reverses these effects (Fig. 6). Of note, the molecular loop affects DLD1 cell migration and the selective tyrosine kinase inhibitor ZD1839 counteracts the EGF-induced phosphorylation of EGFR and ERK. At last, consistent with findings from wound scratch assay, EGF promotes FAK activation that is prevented by ZD1839.

Although not specifically evaluated in this work, the RIZ2-induced increase in EGF secretion could be a very intriguingly point and, at least partially, responsible of some features of CRC pathogenesis and progression that are not yet well dissected and not exclusively restricted to primary tumor cells. Through M2-polarization of tumor-associated macrophages, EGF secreted by colon cancer cells enhances the cancer-driving effect of the tumor microenvironment [52], then promoting immuno-suppression, angiogenesis and neovascularization, as well as stromal activation and remodeling. Moreover, EGF suppresses the expression of LGR5 (a co-receptor for Wnt/β-catenin signaling and marker of both normal intestinal stem cells and cancer stem cells) finely regulated during the adenoma-carcinoma progression, the development and maintenance of CRC-derived metastasis [58, 59]. The finding that *RIZ2* overexpression enhances the EGF/EGFR pathway strongly suggests a not-negligible role of *PRDM2* isoforms in the control of several oncogenic networks involved in CRC encompassing oncogenic pathway crosstalk, tumor microenvironment remodeling, tumor immune-escape, plasticity and stemness. However, the biological role of EGF in CRC is still not completely understood. Although it should act as a negative prognostic factor due to its activation of the MAPK signaling cascade, currently there are no literature studies demonstrating a correlation between serum EGF levels and prognosis in CRC. From a clinical point of view, it would also be interesting to search for a possible correlation between *RIZ2* expression and serum EGF levels in CRC patients as well as their relationship with patient outcomes. The prognostic value of this correlation in larger multi-center studies might enable clinicians to identify subgroups of patients who may benefit from targeted therapy.

Besides, in our opinion, the finding that *RIZ2* overexpression triggers the increase of EGF secretion in the setting of CMS1 background, such as in DLD1 cells, could represent a new element in the effort to better characterize the CRC molecular subtypes and to understand the players able to modify the tumor microenvironment, the immune-escape and, in turn, the optimized sensitivity to first and second line treatments [33, 42].

These results add novel insights on the putative RIZ2 tumor-promoting functions in CRC. However, further investigations are required to explore the RIZ2 molecular mechanisms of action in CRC to exploit the full potential of PRDM2 knowledge for therapeutic applications. For instance, loss-of-function studies might be performed to corroborate our results. We have not used this approach here since it is still difficult to selectively silence *RIZ2* without targeting, at least in part, also *RIZ1* transcript [6, 34]. In this context, one possibility could be to exploit the preferential presence of slightly different 3’ tails in *RIZ1* and *RIZ2* transcripts, as we recently described in lymphocytes [4]. These differences could be useful to selectively target the different *PRDM2* transcripts. Besides, chromatin immunoprecipitation (ChIP) studies coupled with next-generation sequencing on overexpressing cells might provide us the profile of RIZ2 direct downstream targets to further elucidate the molecular bases of its function.

Additional genes and pathways, which have been found to be deregulated in these cells, may be also involved in the RIZ2/RIZ1 imbalance mechanism of action. For instance, *EGFL7* is very highly upregulated in *RIZ2*-overexpressing cells (*FC* = 34.322) (Additional file 1: Table S1). This gene encodes for the epidermal growth factor-like protein-7, which is known to facilitate tumorigenesis and angiogenesis through main cancer drivers from EGFR signaling to integrins, Notch receptor, or lysyl oxidase family members [reviewed in 60]. Interestingly, EGFL7 can also bind to the EGFR at cell membrane and this interaction enhances cell migration of hepatocellular carcinoma cells through FAK phosphorylation [60, 61]. Again, EGFL7 competes with EGF for binding to EGFR [60]. These findings, together with our present work, strongly suggest investigation of this mechanism also in CRC cells overexpressing *RIZ2*.

To assess the clinical relevance of our findings, we performed the analysis of overall survival and disease-free survival of TCGA-COAD patients on GEPIA2. As shown in Additional file 2: Fig S3, the correlation of *RIZ2* expression with these clinical parameters was not statistically significant, probably because of the small number of currently available patients. However, a reduction trend of both overall survival and disease-free survival in TCGA-COAD patients with high *RIZ2* was observed; interestingly, this trend was more evident in patients with MSS status (Additional file 2: Fig S3). Thus, future studies on larger cohorts of patients are warranted to provide the applicability of these results in the management of CRC patients. Besides, an extended analysis of *RIZ2* transcript in further TCGA datasets revealed that it is overexpressed also in other gastroenterological tumors (Additional file 2: Fig S4) thus suggesting a broader role of RIZ2 in cancer biology. At last, analysis of RIZ2 role in CRC cell metabolism and identification of selected domains or functions might provide new insights for their possible clinical use in both diagnosis and therapy of CRC.

### Supplementary Information

**Additional file 1.** RNA-Seq output data of DLD1_EGFP_RIZ2 vs DLD1_EGFP and list of differentially expressed genes according to adjusted p value.

**Additional file 2: Figure S1.** Expression of *RIZ1* (PRDM2-001) and *RIZ2* (PRDM2-003) transcripts in COAD and its subtypes on GEPIA2. Differentially expressed *RIZ1* and *RIZ2* transcripts in COAD samples (A). *RIZ1* and *RIZ2* overexpression in tumor samples compared to normal tissues (B). *RIZ2* expression in COAD subtypes with microsatellite instability-high (MSI-H), microsatellite instability-low (MSI-L), or microsatellite stability (MSS) (C). Histograms represent the *PRDM2* gene expression by qRT-PCR in colon cancer cell lines with MSI (DLD1, HCT116, SW48) and with MSS (SW620). Their expression was verified using two sets of primers recognizing sequences *RIZ1* specific or a common region to both *RIZ1* and *RIZ2* (and indicated as *RIZex8*) (D). **Figure S2.** Immunofluorescence analysis with the anti-EGF antibody (red) on DLD1-pEGFP and DLD1-pEGFP_hRIZ2 positive cells (green). Nuclei are stained in blue. Cells stained with the secondary antibody alone, as negative control, are reported. **Figure S3.** Analysis of Overall Survival and Disease-Free Survival correlation with *RIZ2* (PRDM2-003) expression in TCGA-COAD patients. Analysis was carried out on GEPIA2 platform using either the whole COAD dataset (A) and on patients grouped according to the MSI status (B and C). **Figure S4.** Expression of PRDM2-003 in tumors of the gastroenterological system. GEPIA2 analysis of *RIZ2* (PRDM2-003) transcript in tumor samples compared to normal tissues of the following TCGA datasets: COAD (Colon adenocarcinoma), READ (Rectum adenocarcinoma), ESCA (Esophageal carcinoma), STAD (Stomach adenocarcinoma), LIHC (Liver hepatocellular carcinoma) and CHOL (Cholangio carcinoma).

## Data Availability

The datasets supporting the findings of this study are available from the corresponding author upon reasonable request. The Raw sequencing related to RNA-seq data have been deposited in the EBI ArrayExpress database (http://www.ebi.acuk/arrayexpress) with the following accession numbers: E-MTAB-13103.

## Abbreviations

ChIP: Chromatin immunoprecipitation
CIMP: CpG island methylator phenotype
CIN: Chromosomal instability
CM: Conditioned medium
CMS: Consensus molecular subtypes
COAD: Colon adenocarcinoma
CRC: Colorectal cancer
CSS: Charcoal-stripped serum
DEGs: Differentially expressed genes
ECM: Extracellular matrix
EGFnAb: Neutralizing anti-EGF antibody
EGFR: Epidermal growth factor receptor
FAK: Focal adhesion kinase
FC: Fold change
GAPDH: Glyceraldehydes-3-phophate dehydrogenase
GEPIA2: Gene Expression Profiling and Interactive Analyses2
IPA: Ingenuity pathway analysis
log_2_(TPM + 1): Log_2_ of transcript count per million
MSI-H: Microsatellite instability-high
MSI-L: Microsatellite instability-low
MSI: Microsatellite instability
MSS: Microsatellite stability
PBS: Phosphate-buffered saline
PRDI-BF1: Positive regulatory domain I-binding factor 1
PRDM: PR/SET domain family
RIZ1: Retinoblastoma protein interacting zinc finger protein 1
RIZ2: Retinoblastoma protein interacting zinc finger protein 2
RNA-seq: RNA sequencing
TCGA: The cancer genome atlas
TGFα: Transforming growth factor α
ZD: ZD1839
