# Supplementary material for: RIZ2 at the crossroad of the EGF/EGFR signaling in colorectal cancer
Source: J Transl Med. 2023 Oct 18;21:736. doi: 10.1186/s12967-023-04621-6 (PMC10585774; doi:10.1186/s12967-023-04621-6)
Supplement: Supplementary file 2 — Additional file 2: Figure S1. Expression of RIZ1 (PRDM2-001) and RIZ2 (PRDM2-003) transcripts in COAD and its subtypes on GEPIA2. Differentially expressed RIZ1 and RIZ2 transcripts in COAD samples (A). RIZ1 and RIZ2 overexpression in tumor samples compared to normal tissues (B). RIZ2 expression in COAD subtypes with microsatellite instability-high (MSI-H), microsatellite instability-low (MSI-L), or microsatellite stability (MSS) (C). Histograms represent the PRDM2 gene expression by qRT-PCR in colon cancer cell lines with MSI (DLD1, HCT116, SW48) and with MSS (SW620). Their expression was verified using two sets of primers recognizing sequences RIZ1 specific or a common region to both RIZ1 and RIZ2 (and indicated as RIZex8) (D). Figure S2. Immunofluorescence analysis with the anti-EGF antibody (red) on DLD1-pEGFP and DLD1-pEGFP_hRIZ2 positive cells (green). Nuclei are stained in blue. Cells stained with the secondary antibody alone, as negative control, are reported. Figure S3. Analysis of Overall Survival and Disease-Free Survival correlation with RIZ2 (PRDM2-003) expression in TCGA-COAD patients. Analysis was carried out on GEPIA2 platform using either the whole COAD dataset (A) and on patients grouped according to the MSI status (B and C). Figure S4. Expression of PRDM2-003 in tumors of the gastroenterological system. GEPIA2 analysis of RIZ2 (PRDM2-003) transcript in tumor samples compared to normal tissues of the following TCGA datasets: COAD (Colon adenocarcinoma), READ (Rectum adenocarcinoma), ESCA (Esophageal carcinoma), STAD (Stomach adenocarcinoma), LIHC (Liver hepatocellular carcinoma) and CHOL (Cholangio carcinoma). [file 12967_2023_4621_MOESM2_ESM.pdf]

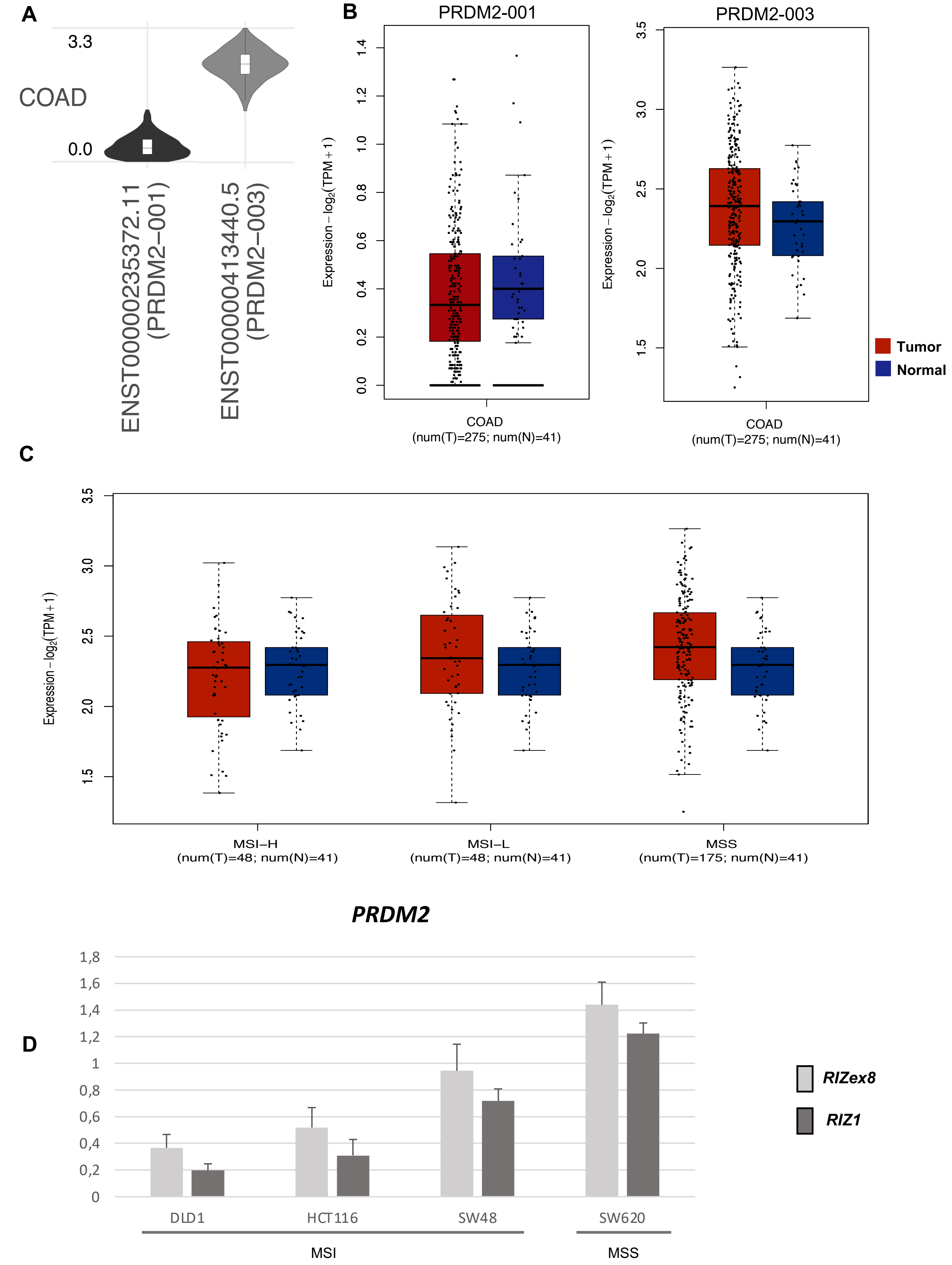

Figure S1. Expression of *RIZ1* (PRDM2-001) and *RIZ2* (PRDM2-003) transcripts in COAD and its subtypes on GEPIA2. Differentially expressed *RIZ1* and *RIZ2* transcripts in COAD samples (A). *RIZ1* and *RIZ2* overexpression in tumor samples compared to normal tissues (B). *RIZ2* expression in COAD subtypes with microsatellite instability-high (MSI-H), microsatellite instability-low (MSI-L), or microsatellite stability (MSS) (C). Histograms represent the *PRDM2* gene expression by qRT-PCR in colon cancer cell lines with MSI (DLD1, HCT116, SW48) and with MSS (SW620). Their expression was verified using two sets of primers recognizing sequences *RIZ1* specific or a common region to both *RIZ1* and *RIZ2* (and indicated as *RIZex8*) (D).

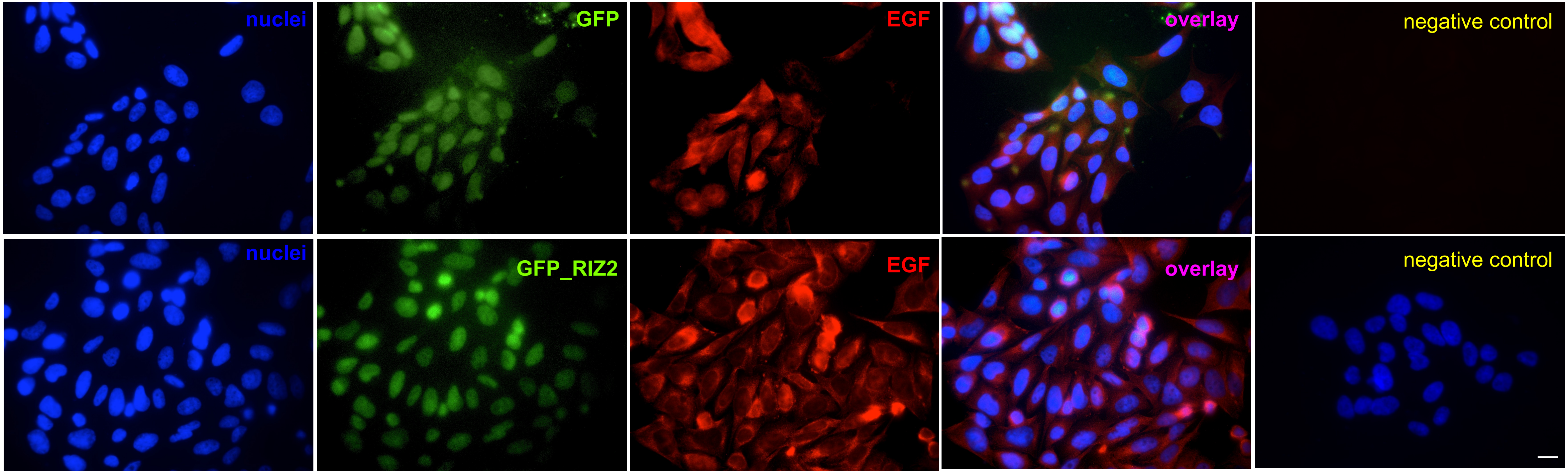

Figure S2: Immunofluorescence analysis with the anti-EGF antibody (red) on DLD1-pEGFP and DLD1-pEGFP_hRIZ2 positive cells (green). Nuclei are stained in blue. Cells stained with the secondary antibody alone, as negative control, are reported.

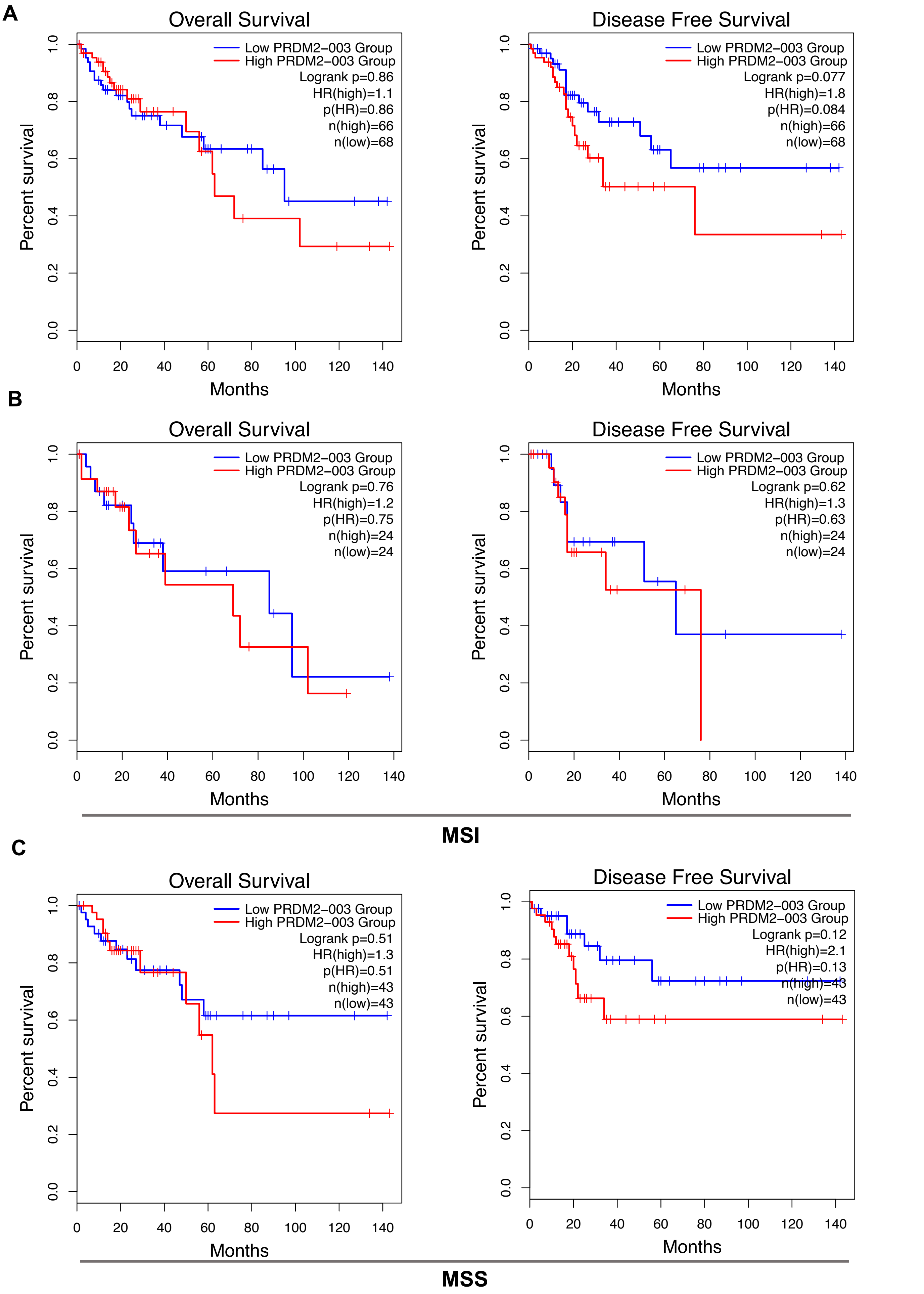

Figure S3: Analysis of Overall Survival and Disease-Free Survival correlation with *RIZ2* (PRDM2-003) expression in TCGA-COAD patients. Analysis was carried out on GEPIA2 platform using either the whole COAD dataset (A) and on patients grouped according to the MSI status (B and C).

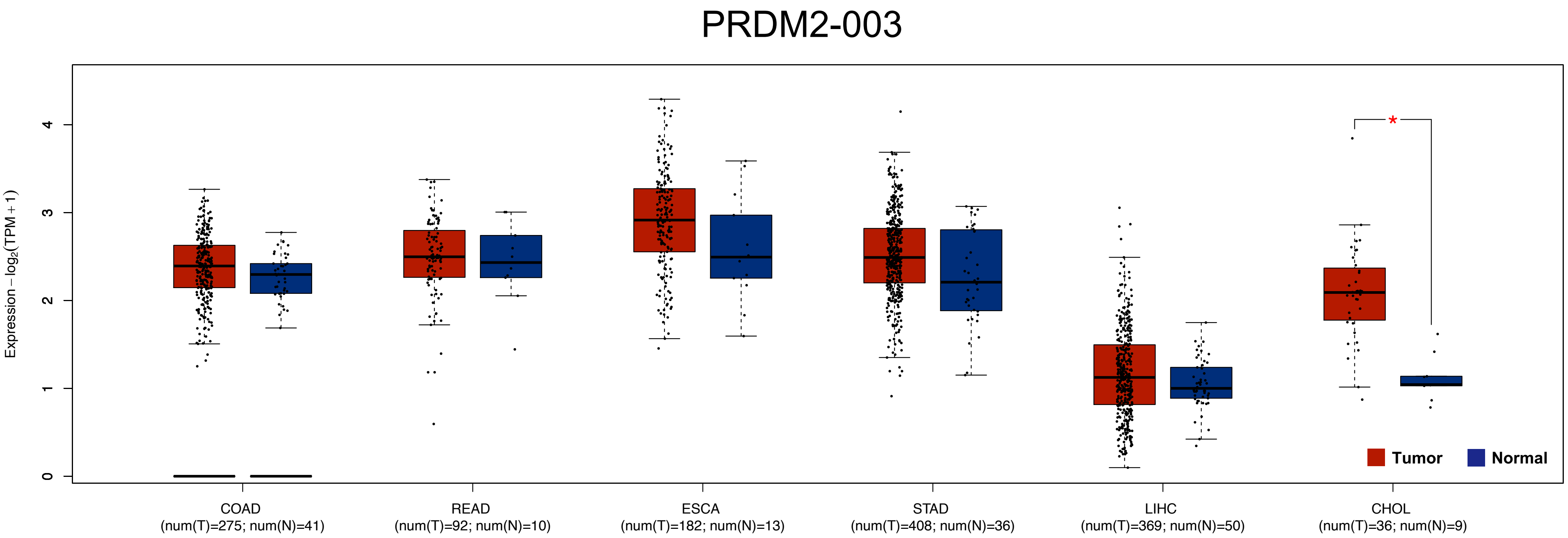

Figure S4: Expression of PRDM2-003 in tumors of the gastroenterological system. GEPIA2 analysis of *RIZ2* (PRDM2-003) transcript in tumor samples compared to normal tissues of the following TCGA datasets: COAD (Colon adenocarcinoma), READ (Rectum adenocarcinoma), ESCA (Esophageal carcinoma), STAD (Stomach adenocarcinoma), LIHC (Liver hepatocellular carcinoma) and CHOL (Cholangio carcinoma).
